# Exploiting the therapeutic potential of contracting skeletal muscle-released extracellular vesicles in cancer: Current insights and future directions

**DOI:** 10.1007/s00109-024-02427-7

**Published:** 2024-03-07

**Authors:** Ana Carolina Pinto, Patrícia Tavares, Bruno Neves, Pedro F. Oliveira, Rui Vitorino, Daniel Moreira-Gonçalves, Rita Ferreira

**Affiliations:** 1 LAQV-REQUIMTE and Department of Chemistry, University of Aveiro, 3810-193 Aveiro, Portugal; 2 iBiMED, Department of Medical Sciences, University of Aveiro, 3810-193 Aveiro, Portugal; 3 CIAFEL, Faculty of Sports, University of Porto and Laboratory for Integrative and ITR, Translational Research in Population Health, 4200-450 Porto, Portugal

**Keywords:** Exercise training, Extracellular vesicles, Proteome, miRNAs, Cancer

## Abstract

The health benefits of exercise training in a cancer setting are increasingly acknowledged; however, the underlying molecular mechanisms remain poorly understood. It has been suggested that extracellular vesicles (EVs) released from contracting skeletal muscles play a key role in mediating the systemic benefits of exercise by transporting bioactive molecules, including myokines. Nevertheless, skeletal muscle-derived vesicles account for only about 5% of plasma EVs, with the immune cells making the largest contribution. Moreover, it remains unclear whether the contribution of skeletal muscle-derived EVs increases after physical exercise or how muscle contraction modulates the secretory activity of other tissues and thus influences the content and profile of circulating EVs. Furthermore, the destination of EVs after exercise is unknown, and it depends on their molecular composition, particularly adhesion proteins. The cargo of EVs is influenced by the training program, with acute training sessions having a greater impact than chronic adaptations. Indeed, there are numerous questions regarding the role of EVs in mediating the effects of exercise, the clarification of which is critical for tailoring exercise training prescriptions and designing exercise mimetics for patients unable to engage in exercise programs. This review critically analyzes the current knowledge on the effects of exercise on the content and molecular composition of circulating EVs and their impact on cancer progression.

## Introduction

Exercise training is well established to have numerous health benefits. In the context of cancer, exercise training is not only safe, but has been shown to reduce incidence and improve survival [1–3]. These findings have motivated researchers to explore the mechanisms by which regular physical activity and physical exercise provide systemic benefits to cancer patients. One proposed mechanism is the release of cytokines produced by contracting skeletal muscle (myokines) into the bloodstream [4–6]. Another potential mechanism is through extracellular vesicles (EVs) released by skeletal muscle (SkM) and their interaction with other tissues [7, 8]. However, research on SkM-derived EVs is currently limited, as only a handful of studies have examined their molecular cargo and the impact of different exercise regimens on their release [9–11]. Furthermore, there is a lack of research investigating the specific role of SkM-derived EVs in cancer development and progression [12, 13].

Early studies on EVs viewed them as a mechanism by which cells get rid of unwanted cellular components. However, in the late 1990s, it was discovered that EVs can transfer proteins, RNA, and even organelles from one cell to another, making them mediators of cell–cell communication [14–16]. EVs are secreted by all types of cells and are present in biological fluids such as blood, cerebrospinal fluid, urine, and saliva [17]. Recent studies have shown that exercise can stimulate the release of EVs from the SkM into the bloodstream, thereby delivering biomolecules to recipient cells [9, 10]. Once EVs attach to these recipient cells, they can affect biological processes by activating cell signaling or passing on their cargo, thereby altering the function and structure of recipient cells [5, 18, 19].

There are three subtypes of EVs: apoptotic bodies (> 800 nm), microvesicles (0.1–1 µm), and exosomes (30–150 nm). Apoptotic bodies are produced by cells during the apoptotic process, while microvesicles, also known as ectosomes or microparticles, arise from the plasma membrane by budding outward. The process of microvesicle formation remains not fully elucidated; nonetheless, it is believed to involve cytoskeleton components such as actin and microtubules, molecular motors (kinesins and myosins), and fusion machinery (SNAREs and tethering factors) [20, 21]. Exosomes, the best-studied small vesicles, are formed by the inward budding of early endosomes that mature into multivesicular bodies and are subsequently released into the extracellular space by exocytosis. Initially considered a means of cellular waste disposal, exosomes have evolved in our understanding to play pivotal roles in cell-to-cell communication, cellular maintenance, and tumor progression [22]. Given the challenges associated with the purification of specific subsets of EVs and the lack of specific markers to distinguish these subsets, the International Society for Extracellular Vesicles has recommended the use of the generic term “extracellular vesicle.” This terminology allows for a more comprehensive approach when dealing with these different vesicular entities [25].

Several mechanisms may be involved in facilitating the transfer of EVs and their cargoes to recipient cells. EVs may initially anchor and posteriorly undergo fusion with the plasma membrane of a target cell. Alternatively, EVs can be internalized through different pathways, including phagocytosis, macropinocytosis, lipid raft–mediated endocytosis, clathrin-mediated endocytosis, or caveolin-mediated endocytosis. Upon endocytosis, EVs may either be directed to lysosomes for degradation or fuse with the delimiting membrane of an endocytic compartment. The latter scenario allows for the release of EV content into the cytosol of the recipient cells (reviewed by [26]). These versatile mechanisms underscore the multifaceted nature of EV-mediated intercellular communication, involving the specific transfer of bioactive molecules such as regulatory proteins and miRNAs, thereby impacting recipient cell functions and phenotype [23, 24].

**Fig. 1:**
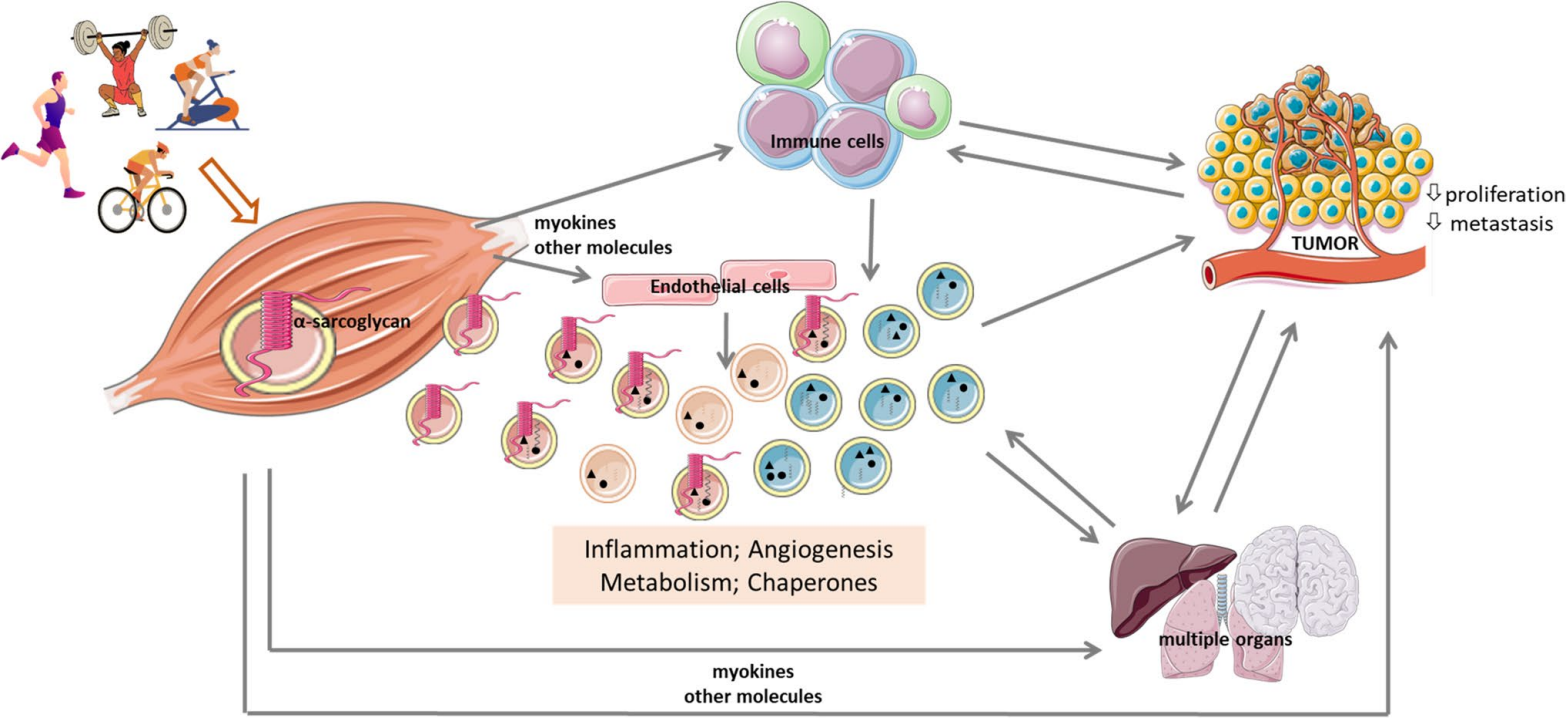
Overview illustrating the impact of physical exercise on circulating extracellular vesicles (EVs), highlighting their origin, destination, and the molecular processes modulated by their cargo following an exercise bout in both trained and untrained subjects. EVs may be released from contracting skeletal muscle (identified by α-sarcoglycan) or other cell populations, particularly immune and endothelial cells, subsequently entering the systemic circulation. These EVs target several organs, depending on their cargo, including specific integrin profiles. Alterations in protein and miR cargo of EVs released post-physical exercise impact several biological processes, notably inflammation, immune profile, angiogenesis, and metabolism. The figure was partly generated using Servier Medical Art, provided by Servier, licensed under a Creative Commons Attribution 3.0 unported license

Although SkM-derived EVs play a critical role in intercellular communication [10, 11, 27–29], the current understanding of their biogenesis and tissue targets for cargo delivery in mammals is still limited. Nevertheless, it was shown that after intraperitoneal injection into mice, EVs derived from the *quadriceps* were detected in cells of at least eight different organs in addition to the SkM, including the brain, liver, heart, lung, gastrointestinal tract, spleen, kidney, and pancreas [30]. This finding highlights the potential of SkM-derived EVs to serve as mediators of inter-organ communication and provides a possible explanation for the systemic benefits of exercise (schematized in Fig. 1). This review aims to critically examine the current understanding of how physical exercise influences the release of SkM-derived EVs, their molecular cargo, and their potential impact on cancer cell proliferation. By integrating current knowledge in this field, we aim to deepen our understanding of the benefits of exercise training in cancer and provide the molecular basis for the development of novel therapies that can mimic these benefits.

## Myokines, exerkines, and extracellular vesicles: Mining on the endocrine potential of skeletal muscle

The SkM accounts for approximately 40% of body mass and serves as an endocrine organ capable of secreting a variety of proteins and peptides (i.e., myokines), lipids, and metabolites that are released by contracting muscles and are essential for mediating some of the systemic effects of exercise [27, 31, 32]. The beneficial effects of exercise include an anti-inflammatory role and positive effects on glucose and lipid metabolism, which appear to be mediated by myokines [33, 34]. Hundreds of myokines (more than 650 proteins) are expressed and released by the SkM [35, 36] and have been termed “myokinome” [37, 38]. Some of these myokines are upregulated by physical exercise, the so-called exerkines, which play a key role in mediating inter-organ communication for the purpose of energy supply, among other functions [38]. This communication is facilitated by ligand-receptor binding complexes [24]. Among these exerkines, FGF-21, IL-6, IL-15, irisin, and BDNF have low concentrations in SkM under resting conditions but increase significantly during muscle contraction [39]. Both continuous moderate-intensity walking and high-intensity intermittent walking have been shown to increase systemic levels of IL-6, with peak levels reached immediately after exercise, followed by a sustained elevation after 4 h and a subsequent gradual decline [40]. Similarly, both moderate- and high-intensity intermittent cycling have been found to increase circulating IL-6 levels [41]. In fact, IL-6 from SkM increases exponentially immediately after an exercise bout, triggering an anti-inflammatory response characterized by increases in IL-1ra and IL-10, while TNF-α is dampened [42]. Furthermore, in endurance-trained individuals, acute exercise has been shown to increase other cytokines such as IL-5 and IL-10. Notably, even in sedentary individuals, IL-10 increases after acute exercise [43].

Most of the exerkines are believed to travel through the body enclosed in EVs [44–46]. Indeed, a significant enrichment of proteins entrapped in EVs was found among the 300 proteins that increased in the bloodstream after a 1 h cycling in healthy individuals [25]. When mass spectrometry (MS)-based approaches were used to identify proteins in EVs, a substantial proportion of these proteins was not previously known to be released into the bloodstream or to contain a signal peptide sequence, as shown by the SignalP server. This suggests an alternative mechanism by which myokines are transported by EVs to recipient cells, mediating organ crosstalk [24]. Indeed, several exerkines, including FGF-21 and GDF-15, are already catalogued in ExoCarta and Vesiclipedia, two databases that compile biomolecules identified in EVs [45].

Exercise training has been reported to regulate both the levels and the molecular cargo of EVs circulating in the bloodstream. For example, Fruhbeis and colleagues [7] demonstrated a 2.7-fold increase in EVs in the venous blood of healthy volunteers who exercised regularly (at least 3 training sessions per week), immediately after a single bout of exhaustive cycling exercise, returning to baseline after 90 min. Furthermore, investigations with human subjects at cardiometabolic risk who underwent exercise stress testing showed an increase in plasma levels of EVs, but not in their size [47]. Nonetheless, not all studies have echoed similar outcomes in terms of EV content or size after a single bout of exercise. For instance, physically active healthy volunteers submitted to a single eccentric exercise that resulted in muscle damage showed no changes in the concentration and size distribution of plasma EVs. However, there was a negative association between the changes in EV release before and after exercise and creatine kinase activity, a biological marker of muscle damage [48]. Similarly, Just et al. [49] found no alterations in the concentration and size of EVs isolated from the plasma of physically active healthy volunteers 1 h after a single bout of blood flow–restricted resistance training, an unfamiliar modality for these participants. Interestingly, following a single exercise session of moderate intensity, a subtle rise in the total plasma EV count was observed in healthy sedentary subjects, while trained individuals showed no such increase within 2 h post the conclusion of the aerobic exercise [50]. In this context, subjects with type 2 diabetes displayed no changes in the levels of SkM-derived EVs after 1 h of exercise on an ergometer compared to non-diabetic obese subjects [51]. These SkM-derived EVs were identified by flow cytometry as lactadherin-binding, phosphatidylserine-positive particles expressing β-sarcoglycan. In contrast, after acute moderate exercise on a treadmill (at 60% of their VO2 max), a decrease in the release of EVs, predominantly in the microvesicle size range, into the bloodstream was found in moderate-weight and obese subjects [52]. The authors noted that the content of circulating EVs depended on body mass index, insulin sensitivity, and gender. In addition to variations in the levels of circulating EVs, it remains uncertain whether the proportion of SkM-derived EVs increases beyond the baseline of 5% after an exercise bout [10], whether in physically active or sedentary individuals. The influence of sex on EV content and profile was further investigated by subjecting both men and women who completed a 5 day simulated military operational stress protocol, involving daily physical exertion, sleep deprivation, and caloric restriction. Notably, EV concentration decreased in women following 48 h of sleep and caloric restriction while remaining stable in men. Both men and women exhibited an increase in EV size, and the proportion of EVs expressing α-sarcoglycan also increased in both sexes, though women displayed a higher content of SkM-derived EVs [53]. These findings underscore the importance of considering biological variables, such as sex, in EV research.

Most circulating EVs originate from cells of the immune system, endothelium, and platelets and persist after exercise. This is underscored by the relatively low abundance of muscle-specific markers in EVs, such as α-sarcoglycan and miR-206, as opposed to the elevated levels of immune cell markers such as CD14, a marker of monocytes/macrophages [10, 52, 54]. Indeed, a comprehensive EV-phenotyping analysis was conducted to investigate the cellular origin and potential subtypes of EVs isolated from the plasma of healthy individuals subjected to an incremental cycling test until exhaustion. The analysis of circulating EVs using a multiplexed flow-cytometry platform identified lymphocytes (CD4, CD8), monocytes (CD14), platelets (CD41, CD42, CD62P), endothelial cells (CD105, CD146), and antigen-presenting cells (MHC-II) as the primary parental cells contributing most to the EV-mediated effects of exercise [55]. Bryl-Gorecka et al. [56] used Olink technology to characterize the proteomic landscape of EVs isolated from plasma samples of volunteers with an average age of 58 years, a subset of whom had cardiovascular pathologies and/or were under medication. These individuals were subjected to 10 min of progressively increasing intensity stationary bicycle exercise, with samples collected 1 h later. Their results showed significant changes in 58 proteins within EVs compared to pre-exercise levels. The cargo contained in these EVs came from different cell types, with immune and endothelial cells making the largest contribution. Of note, only a dozen SkM-derived proteins, including myoglobin and follistatin, were modulated by acute exercise. Similarly, Kobayashi et al. [57] demonstrated that a proportion of circulating EVs, isolated from the plasma of young males after 8 weeks of high-intensity interval training, originated from various tissues and cells, including hepatocytes and adipose tissue. The relatively low proportion of EVs derived from the SkM is interesting, considering that the SkM contributes significantly to total body weight. However, SkM exhibits interstitial space and a vascular endothelial barrier between myofibers and circulation. The extent to which EVs derived from myofibers reach the circulation remains incompletely understood, as highlighted previously [54]. This also raises questions about the mechanisms by which SkM-derived signaling molecules exert their systemic effects, as well as the cellular sources of EVs released after exercise. Moreover, it is unclear whether these cellular sources and molecular mechanisms are the same in physically active and sedentary individuals submitted to a bout of exercise.

It is important to acknowledge that determining the proportion of SkM-derived EVs to total EVs in circulation poses several challenges. First, the presence of markers specific to the SkM origin, such as β- or α-sarcoglycans, integral components of the dystrophin-glycoprotein complex, cannot be guaranteed in all SkM-derived EVs [17], making it difficult to definitively confirm EVs originating from SkM contractile activity. Second, α-sarcoglycan has been detected not only in SkM but also in cardiac muscle [58]. Third, intramuscular injections of fluorescently labeled EVs or genetic manipulation, which are excellent options in rodent models, are impractical in human studies [17]. Despite the difficulties in studying SkM-derived EVs in vivo, several in vitro studies have clearly demonstrated that both myoblasts and myotubes are capable of releasing EVs whose biological role in recipient cells is unclear [59, 60]. Therefore, there is a need for alternative methodological approaches to reliably determine the contribution of SkM-derived EVs in in vivo studies.

Apart from their origin, understanding the destination of exercise-induced EVs is crucial to gain deeper insights into the molecular mechanisms underlying the systemic effects of physical activity and exercise. The organ-specific targeting of EVs appears to be facilitated by adhesion proteins, which have been reported to increase in the bloodstream after exercise. Interestingly, one of these adhesion proteins, integrin beta 5 (ITGB5), has exhibited presence in EVs secreted from the limbs of mice exercising on a treadmill and found incorporated into the liver cells treated with these EVs [24]. Another adhesion protein, ITGA2B, known for its pivotal role in the coagulation cascade, showed an increase in EVs collected from the plasma of physically active healthy volunteers after a bout of resistance training with restricted blood flow [49]. Therefore, it is plausible to suggest that integrins play a pivotal role in the complex systemic targeting of EVs toward specific organs during exercise. In these target organs/cells, EVs release diverse protein cargo, including glycolytic enzymes [24] and proteins involved in the uptake of long-chain fatty acids [51]. The potential of these proteins to influence the metabolic rate of recipient cells is a noteworthy aspect. In response to the high energy demands of exercise, these metabolic proteins are more abundant in EVs. A considerable subset of proteins modulated by, for example, 10 min cycling, identified in EVs extracted from the plasma of older adults, some of whom have cardiovascular disease, appears to be related to other biological processes such as inflammation, angiogenesis, and coagulation [56]. This intricate array of proteins illustrates a complex interplay that forms the basis for the multiple systemic effects evoked by physical exercise.

## The impact of exercise on the miRNome of extracellular vesicles

Extracellular microRNAs (miRs) are usually found either bound to protein complexes, associated with high-density lipoproteins or encapsulated in small EVs [61]. The amount and composition of the miR cargo likely depend on the modality and intensity of exercise [27, 62], similar to the proteome of EVs. Indeed, total miR content in plasma EVs was reported to increase by almost 70% after acute exercise, and 13 miRs were significantly enriched by exercise, returning to baseline levels after a recovery period of 2 h. These miRs shown to be modulated by acute exercise include miR‐10b‐5p, miR‐222‐3p, miR‐23a‐3p, miR30a‐5p, miR484, miR‐652‐3p, miR‐92a‐3p, and miR991‐5p; however, only miR‐145‐5p and miR‐424‐5p are strongly expressed in muscle cells, while the other miRs originate in immune and endothelial cells. Moreover, an increase in miR-126 plasma levels was observed after a 4-h cycling at 70% of the anaerobic threshold, which persisted throughout the duration of the exercise. The blood levels of miR-133 and miR-126 were also shown to be markedly increased in middle-aged male marathon runners [63]. In a study with 13 male swimmers participating in a fatiguing 1500 m freestyle swimming session at the speed of their best-recorded performance, the miR profiles of circulating EVs underwent significant changes, marked by elevated levels of miR-144-3p, miR-145-3p, miR-509-5p, miR-891b, and miR-890 [64]. In healthy, untrained subjects aged 18 to 30 years, a significant decrease in miR-31 was observed 24 h after a mildly muscle-damaging exercise bout in the form of a plyometric jump followed by downhill running compared to baseline levels [65]. Although it did not induce alterations in the amount of circulating EVs, resistance training with restricted blood flow was shown to upregulate the expression of hypoxia-inducible miR-182–5p, which was packaged into EVs. This specific miRNA has been shown to be capable of enhancing HIF-1α signaling, protecting cardiomyocytes from hypoxia-induced apoptosis, modulating glucose utilization in SkM, and increasing angiogenesis in vitro [49]. In a recent study, the miR cargo from EVs isolated from biopsies of the vastus lateralis after 1 week of concurrent aerobic and resistance exercise was shown to contain miR-1, miR-133, miR-206, miR-486, and miR-499 as major SkM-specific miRs [65]. Interestingly, in a cohort comprising both young male and female subjects participating in a 60 min cycling session at 70% VO2peak, no correlations were found between EVs and SkM miR expression. This suggests that EV miR content may not represent SkM miR expression. Furthermore, this study identified sex-specific differences in the miR response to an acute bout of endurance exercise, particularly concerning miR species associated with mitochondrial metabolism and angiogenesis [66]. In a clinical study, subjects underwent 40 min of vigorous-intensity aerobic exercise (80% VO2 max), and SkM-derived EVs were immune-captured with an antibody against α-sarcoglycan. These α-sarcoglycan-positive EVs were highly enriched in SkM-specific miR-206, highlighting its role in mediating the effects of exercise. A significant increase in other SkM-specific mRNAs, such as miR-181a-5p and miR-133b, was also detected in α-sarcoglycan-positive EVs after exercise. However, α-sarcoglycan + EVs account for only about 5% of the total EV population [10], as discussed previously. Although the presence of SkM-derived EVs in circulation is low, both cytofluorimetric data and myo-miR quantifications unveiled an elevation in SkM-related signals transported by EVs in the bloodstream following a single bout of exercise in sedentary subjects. Notably, the intensity of exercise plays a crucial role in determining the systemic increase in EV-miRs, with a higher elevation observed in exhaustive incremental exercise compared to acute aerobic exercise [50].

Once internalized into target cells, EV-miRs can regulate gene expression and promote physiological effects [67, 68]. For instance, miR-31 is known to transiently suppress translation of satellite cell activator Myf5 mRNA and maintain satellite cells in a quiescent state [65]. Pathway analysis revealed that SkM-specific miRs enclosed in EVs isolated from vastus lateralis after short-term concurrent exercise training (referring to miR-1, miR-133, miR-206, miR-486, and miR-499) modulate the inflammatory response [69]. From these, miR-206 and miR-486 were also associated with SkM hypertrophy and regeneration [70]. Exercise upregulated miR‐222‐3p, miR‐30a‐5p, and miR‐10b‐5p and have been associated with the regulation of myoblast proliferation and myofiber formation [19]. Mechanical overload triggers the release of EVs containing muscle-specific miR-1, which seem to be preferentially taken up by epidydimal white adipose tissue, where it promotes lipolysis [71]. Blood flow–restricted resistance exercise promoted the upregulation of six miRs involved in protein translation, Akt/mTOR signaling, and NF-kB activation [49]. Moreover, the beneficial effects of miR-containing EVs isolated from interval-trained muscles were found on glucose tolerance in sedentary mice [72].

Overall, the miRs carried by EVs are responsive to exercise-induced muscle contraction and play a role in modulating the functionality of recipient cells [73]. The secretion of EVs and their cargo is influenced by various factors such as the mode, intensity, and duration of exercise programs [10, 40, 43, 74]. For example, exhaustive incremental exercise leads to a more pronounced and faster increase in SkM-derived miRs compared to acute aerobic submaximal exercise [50]. The health status of the subjects who participated in these studies is also a key factor, as it can strongly influence miR profiles in circulating EVs. In fact, athletic and sedentary men, even when submitted to the same exercise protocol, exhibit different miR profiles in EVs [75]. The precise stress signals that trigger changes in SkM and other organs and lead to alterations in miRs and other molecules in circulating EVs are still largely unknown.

## Effect of skeletal muscle–derived EVs in cancer development and progression

The benefits of exercise in a cancer setting include normalization of the tumor’s vascular system and metabolism, alteration of the systemic immunological profile, and improvement of tissue immune cell surveillance [76]. These anticancer effects are hypothesized to be mediated by contractile factors secreted by SkM. In vitro studies have shown that human serum from subjects who performed two high-intensity endurance cycling sessions affected the proliferative and microtumor-forming capacity of breast and prostate cancer cell lines [77]. These authors also demonstrated that the exercise-induced effects on cancer cell proliferation were due to the high intensity of the training session and not to the training duration. Devin et al. [78] treated colon cancer cells with serum samples from colon cancer survivors who had completed either an acute high-intensity interval training or a chronic exercise program with 12 high-intensity interval training sessions. Their results showed that serum obtained immediately after high-intensity interval exercise significantly decreased the number of colorectal cancer cells, while no significant effect was observed 120 min after training. Serum from women with breast cancer and from healthy women obtained during and immediately after an exercise session also showed promising results. In vitro experiments showed a significant reduction in the viability of hormone-sensitive and hormone-insensitive breast cancer cells by about 10%. The serum of exercised women also reduced tumor formation by 50% when MCF-7 breast cancer cells (hormone-sensitive) were inoculated into NMRI-Foxn1nu mice. One proposed mechanism to explain the tumor-suppressive effect of exercise is the activation of β-adrenergic signaling, which appears to modulate the Hippo pathway [79]. However, not all exercise programs have the same effect. Despite improvements in cardiorespiratory fitness (VO2 peak) and muscle strength after 6 months of exercise training, no significant changes in breast cancer cell viability were observed in vitro after incubation with the serum of these trained breast cancer patients [80]. In another study, serum from male subjects cycling at increasing intensity for 60 min resulted in a 31% inhibition of the growth of the prostate cancer cell line LNCaP, and pre-incubation prior to subcutaneous injection into SCID mice caused a delay in tumor formation [81]. In addition, treatment of lung cancer cells with serum collected 5 min, 1 h, and 24 h after exercise on a cycle ergometer significantly inhibited cell survival and proliferation. Interestingly, the human post-exercise serum used for cell treatments only decreased the viability of cancer cells without affecting the viability of normal cells [82]. These results suggest that some of the biomolecules that enter the bloodstream after exercise may reduce the proliferation rate and survival of tumor cells.

Regarding the potential anticancer effect of EVs derived from the plasma of exercised subjects, only one preclinical study is known to have demonstrated the tumor-suppressive effect of such EVs. In this study by Sadovska et al. [12], regular injection of exercise-induced EVs in tumor-bearing rats resulted in a reduction of primary tumor growth by approximately 35% and a possible delay in the development of lung metastases. When analyzing the cargo of exercise-induced EVs, these authors found upregulation of genes encoding proteins involved in metabolic processes, such as *Notum* (palmitoleoyl-protein carboxylesterase), *Pctp* (phosphatidylcholine transfer protein), and *Cyp4b1* (cytochrome P450, family 4, subfamily b, polypeptide 1). In addition, the molecular chaperones *Dnajb5* and *Hspa5* were identified, which are involved in protein maturation and cell survival under stress conditions. The cargo also contained molecular players associated with inflammation (*Ltb4r2* and *Alox5*), T-cell development (*Zbtb1*), and germinal center B cells (*Fcrlb*). Despite the small sample size, this study sheds light on the molecular composition of exercise-induced EVs and their potential role in inhibiting tumor growth and metastasis in vivo (Fig. 1). Further research in this area is warranted to investigate the therapeutic potential of exercise-induced EVs in cancer treatment.

## Unexplored dimensions in understanding the role of EVs in exercise setting

EVs have been proposed to act as mediators of exercise-induced health benefits by acting as cargo carriers or intermediaries facilitating inter-organ communication with beneficial outcomes. Nevertheless, before EVs can be considered plausible therapeutic strategies, several questions need to be addressed.

The question arises whether EVs represent the plasma fraction that most accurately reflects the effects of physical exercise and should therefore be the focus of exercise mimetics development studies. Piccirillo [44] suggested that the development of an “exercise pill” capable of reproducing the multiple benefits of exercise on different organs is impractical due to the complex and interrelated pathways affected by exercise and the influence of individual genetics on the holistic effects of exercise. As an alternative, he suggested researching selected myokine-based drugs that target specific pathways and could help patients who are unable to exercise for various reasons. However, there is still no evidence for the therapeutic use of myokine-based drugs.

An interesting topic that remains to be explored is the possible presence of organelles, particularly mitochondria, in exercise-induced EVs. It is possible that these EVs contain functional mitochondria that could contribute to the anti-inflammatory effects of exercise. This hypothesis parallels the immunomodulatory effects observed in mesenchymal stem cells (MSCs) in conjunction with their mitochondrial cargo [83]. The proteomics analysis of SkM-derived EVs from exercised mice revealed an enrichment of proteins associated with mitochondrial biogenesis [84]. Moreover, intact and functional cell-free mitochondria have been identified in the bloodstream and appear capable of re-entering cells. Both flow cytometry and proteomics data corroborate that, at least in part, cell-free mitochondria are encapsulated within EVs [85]. Recently, it has been demonstrated that mitochondria are eliminated in large EVs via the endosomal pathway when lysosomal degradation is impeded [86]. Therefore, investigating the presence and functional role of mitochondria in exercise-induced EVs could provide valuable insights into the mechanisms underlying the beneficial effects of exercise.

Moreover, it remains unclear how SkM contractile activity specifically influences the molecular profile of EVs. Our understanding of EVs from SkM fibers is primarily derived from monocultures of proliferating myocytes and, to a lesser extent, differentiated myotubes. Proliferating myocytes may not exhibit behavior identical to the terminally differentiated fibers predominant in SkM tissues. While monocultures offer a straightforward approach to studying SkM fiber–derived EVs, they lack the cellular heterogeneity and extracellular matrix found in tissue environments. As an alternative model, SkM explants have been proposed. In such explants, various cell types beyond myofibers may contribute to the secretion of EVs [54]. However, investigating the influence of exercise programs on the EV secretion profile using SkM explant models, particularly those derived from humans, presents notable challenges, and the evaluation of systemic effects may be limited.

The secretory activity of the SkM appears to be influenced by exercise-induced pH changes. During submaximal cycling followed by a 60-min passive recovery period, the pH of the SkM is known to remain between 7.08 and 7.16 [87]. In contrast, the pH of the SkM can drop to as low as 6.42 during maximal exercise [88]. Although this pH drop is not as severe as shown in in vitro models, there is evidence that maximal intensity and intermittent exercise lead to a greater drop in SkM and plasma pH than moderate-intensity, continuous exercise [89]. These pH variations could explain, at least in part, the effects of different exercise regimens on the molecular profile of circulating EVs. Further research is needed to investigate the relationship between exercise intensity, duration, pH changes, and the resulting molecular profile of exercise-induced EVs.

**Fig. 2:**
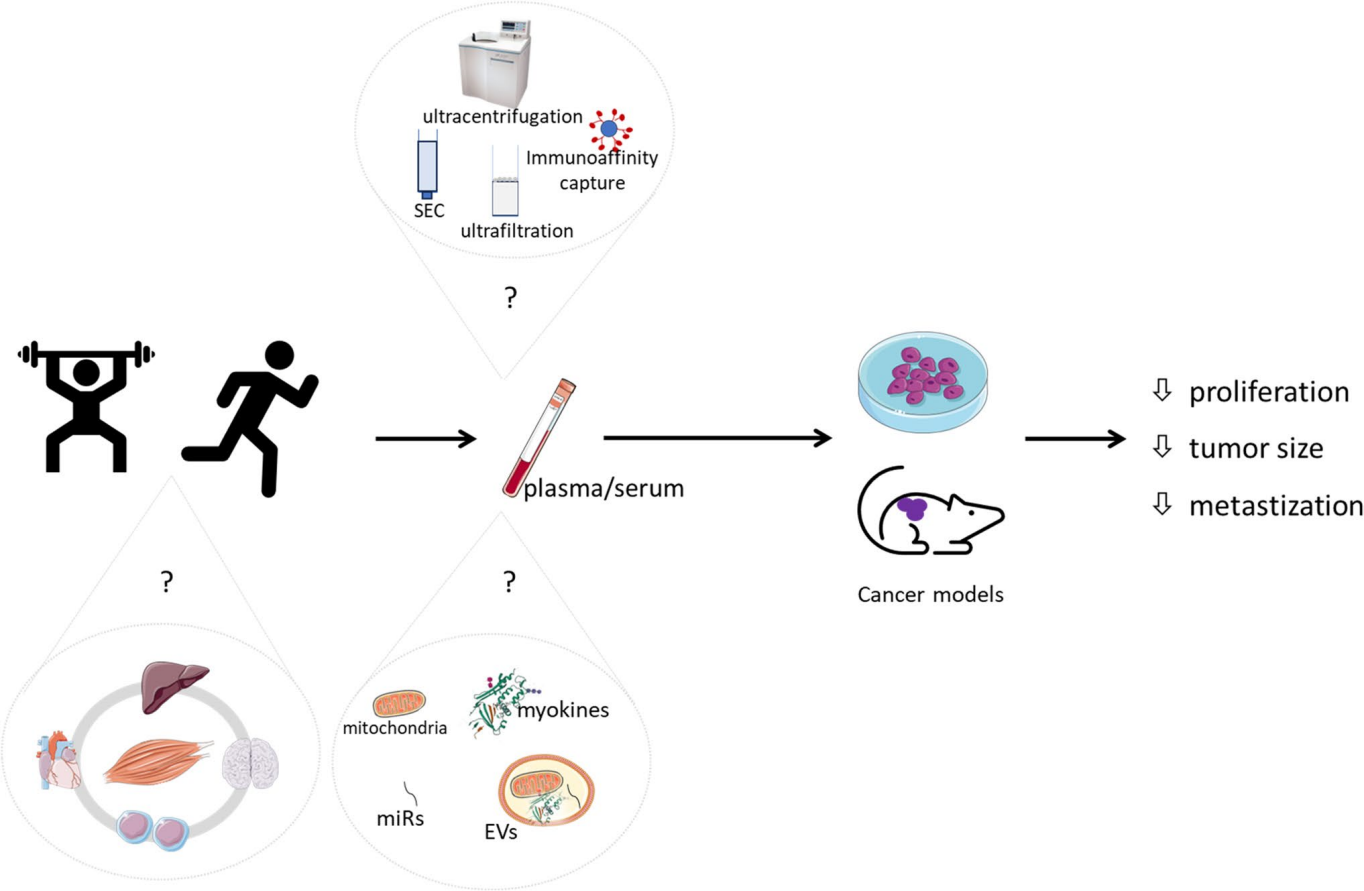
Current challenges in investigating the putative anticancer effects of blood-derived plasma or serum from exercised individuals. The figure was partly generated using Servier Medical Art, provided by Servier, licensed under a Creative Commons Attribution 3.0 unported license. Legend: EVs, extracellular vesicles; SEC, size-exclusion chromatography

Another important question is which exercise regimen produces the most favorable molecular profile of EVs in cancer patients. Most studies report either positive or no anticancer effects of EVs isolated from trained subjects; however, these results are primarily from in vitro studies, with only one study using an animal model [12]. Therefore, further in vivo studies are essential to define an exercise mimetic based on EV profiles. These studies should also strive to establish a consensus on the EV cargo profile within the FITT paradigm (frequency, intensity, time, and type) of exercise training, as has been proposed [70], tailoring considerations to each specific clinical condition. Importantly, there is currently no evidence to support the assumption that the health benefits of an exercise-induced EV profile apply uniformly to all diseases, including various types of cancer. While it is generally accepted that every exercise bout matters against the development and progression of cancer [90], it is still unclear which specific exercise parameters maximize the benefits mediated by EVs. There are several methodological issues to consider when comparing the effects of exercise programs. One of these issues is the timing of blood sampling, as differences in timing may lead to discrepancies between studies. The physiological changes induced by acute training may persist for up to 24–48 h after the end of training but do not necessarily represent an adaptive response to long-term training. Some metabolic effects and purported health benefits of exercise, such as lowering blood pressure or improving circulating lipoprotein profile, may be attributed to the biological consequences of the most recent exercise session rather than true adaptations from long-term training (reviewed in [91]). Furthermore, the use of distinct methods to isolate, quantify, and characterize EVs in different studies may complicate the interpretation of the effects of specific exercise programs [92]. Ultracentrifugation-based methods remain the gold standard for isolating EVs. Nevertheless, alternative methods have emerged to address inherent challenges, including the need for large starting sample volumes, time-consuming procedures, copelleting of high molecular mass protein complexes and lipoproteins, and the limitation in precisely enriching specific EV subpopulations. These alternatives, based on isolation by size, immunoaffinity capture, and exosome precipitation, have been developed. Despite these innovations, they often fall short of achieving exclusive EV isolation, leading to complex mixtures comprising EVs and other extracellular space components [20, 21]. Figure 2 provides an overview of the challenges encountered when investigating the anticancer effects of exercise training using blood-derived fluids, and their constituents, namely EVs.

## Concluding remarks

Despite the growing recognition of the central role of EVs in mediating the therapeutic benefits of exercise training in diseases such as cancer, several important questions remain unanswered, hindering the translation of evidence on exercise-induced EVs into clinical applications. To our knowledge, only one in vivo study has investigated the anticancer potential of EVs isolated from blood-derived samples after exercise [12]; however, the use of a limited animal cohort hampers robust validation of the role of EVs in mediating exercise effects. Furthermore, the extent of the contribution of SkM to the composition of EVs needs to be better understood. While the proteome cargo of EVs released after exercise suggests a limited influence of SkM secretory activity on the overall pool of circulating EVs, specific miRs, such as miR-206, have been identified in EVs released by contracting SkM [69] and found to increase after exercise [10]. This underscores the significant role of miRs in shaping the molecular profile of circulating EVs during exercise, despite the apparent limited influence of SkM secretory activity on the overall EV pool. Nevertheless, it is plausible that the impact of exercise on this profile could be indirect, potentially influencing the secretory activity of other cells, particularly endothelial and immune cells. While there is general agreement on the changes in the content, size, and/or cargo of EVs following acute exercise (in comparison to rest), the impact of chronic exercise is less clear. This complexity is further heightened in individuals with underlying pathological conditions. Therefore, future studies are needed to envision the definition of exercise regimens tailored to individual health status, age, gender, and other relevant characteristics, with the aim of optimizing the amount, size, and molecular cargo profile of EVs in the bloodstream after exercise. By deciphering the molecular and functional properties of exercise-induced EVs, we can pave the way for the development of innovative therapeutic strategies based on specific molecular compounds that mimic the benefits of physical exercise for those patients who are unable to engage in regular physical activity due to various health limitations.

## Data Availability

Not applicable.
